# Pyæmia and Septicæmia, in Their Surgical Aspects*Read before the Virginia Society of Medicine, September 4, 1895.

**Published:** 1895-09

**Authors:** J. McFadden Gaston

**Affiliations:** Atlanta, Ga.


### Southern Medical Record.

A MONTHLY JOURNAL OF MEDICINE AND SURGERY.

VOL. XXV. ATLANTA, GA., SEPTEMBER, 1895. No. 9.

### Original Articles.

### PYÆMIA AND SEPTICÆMIA, IN THEIR SURGICAL ASPECTS.*

BY J. McFADDEN GASTON, M. D., ATLANTA, GA.

During the past decade my views have been presented to the profession on sundry occasions touching the important role of septic processes in the physical organism. There is nothing, in my estimation, connected with pathology, of more pervading and far reaching influence, than the grave consequences of general contamination.

The insidious progress of infection of various forms in undermining and disintegrating the vital organs, appeals to the medical philosopher and the surgical pathologist for the most profound consideration. A thorough comprehension of the normal condition of the various structures which enter into the composition of the different organs of the body is a prerequisite for understanding their disorders and diseases, and some familiarity with biology and histology is, therefore, essential for entering upon an investigation of septic troubles.

The general term, blood poisoning, has been applied to a great variety of contaminations of the organism, and while pyæmia is regarded as the result of the absorption of pus into the vascular system, septicæmia implies infection from different sources. It is not expected that my views on these departures from the normal condition of the organization shall

*Read before the Virginia Society of Medicine, September 4, 1895.

correspond entirely with the established pathological tenets of our text-books, or with the doctrines recognized as orthodox by my intelligent colleagues who constitute this society. But the agitation connected with the principles involved in pathology has always led to practical results of great importance, and my object in this investigation is to elicit discussion on the part of the members of this body, of the points presented for your consideration. I do not claim to be able to instruct you in regard to these matters, but hope to receive useful knowledge from those who have had experience in treating this class of cases. It is not simply commentation I seek, but it is criticism, which may prove instructive to myself and others, that is desired in discussing this paper.

In overhauling my individual experiences as to pyæmia and septicæmia and comparing them with the observations of others touching their medical aspects, I have become satisfied that there are many features of their surgical aspects which can appropriately be embodied in a paper at this time. It has occurred to me, therefore, that the present occasion may prove opportune for bringing out this phase of the subject; and my contribution upon pyæmia and septicæmia to the "Twentieth Century Practice of Medicine," not being expected until that publication is far advanced, I trust this may prepare the readers of that work for a better comprehension of the principles involved in this investigation.

The practical bearings of pyæmia and septicæmia have cropped out in various forms connected with surgical work, and more especially in what has been designated as antiseptic surgery. This has been developed conspicuously by Sir Joseph Lister's process of treating surgical cases with special regard to the presence of septic features in the structures.

His conception of the surroundings of a patient being attended with contaminations extending to the field of operation led to various antiseptic devices, and especially to securing cleanliness. Out of the vast array of elaborate dressings which were inculcated, little remains to-day of Listerism excepting the scrupulous avoidance of filth of every kind, constituting asepsis. In so far as the experience of Mr. Lister, in subsequent years may guide surgeons in the use of germicidal agents for operations, all are aware that he now prefers carbolic acid washes to those of corrosive sublimate, and that they are employed with great caution.

He confines the application of antiseptics to septic conditions of the parts involved and adheres mainly to cleanliness in the physiological state of the tissues.

Much credit is due to Sir Joseph Lister for drawing the attention of surgeons to the importance of avoiding contamination by filth of all kinds, but the large following he had formerly in antiseptic surgery has gradually decreased, until the vast majority of surgeons and gynæcologists of the present day rely exclusively upon aseptic measures in normal tissues.

Among those who have taken a part in the study of bacteriology as allied to surgery, I have watched with great interest the development of the germ theory of the origin of diseases, and yet have not been so carried along with the current of opinion as to ignore entirely the fact that thus far it has not met all the requirements for comprehending pathological conditions.

Without discrediting scientific experimentation, there is quite sufficient evidence, that grave mistakes have been made in the mode of investigating the relations of cause and effect, connected with the presence of a microbian element in the different parts of the organism. That some diseases are uniformly accompanied with bacilli of a definite kind, does not suffice, to give a final decision as to the etiological factor in these diseases; and much less is it a conclusive indication for the therapeutic measures which are required for the relief of such a disorder.

The prestige of antiseptic surgery was such ten years ago, that a surgeon who dared to neglect the recourse to germicidal applications in his operations was styled an old fogy and unworthy of recognition by those of the advanced antiseptic practice. The zeal in propagating Listerism reached such a point that a surgeon who might have an untoward result without observing its tenets, would have had the testimony of its defenders against him in a suit for malpractice, and he would have been placed in a most embarrassing attitude before the medical world. But a change has come over the spirit of the surgeon's dream at the present day, and instead of being on the defensive, the opponent of germicidal washes in normal structures, is now occupying the offensive position. The best class of bacteriologists to-day are supporting him in condemning all poisonous applications, excepting only those cases already contaminated, which call for correction by antiseptics. In these alone is the so-called antiseptic surgery warranted.

The high authority of the Johns Hopkins Faculty, headed by the distinguished bacteriologist, Dr. Welch, is arrayed against the employment of any germicidal or prophylactic solutions in operating upon normal structures, and sterilized water only is used in washing fresh cut surfaces. In the very instructive discussion upon radical operation for hernia before the Southern Surgical and Gynæcological Association at Louisville, Dr. Howard A. Kelly criticized the technique of a distinguished colleague, who thought it proper "to conduct the entire operation under irrigation, with a weak sublimate solution." "I would urge him," he stated, "to abandon the use of solutions of the bichloride of mercury. The value of the inhibitory power of this drug over germ life is more than counterbalanced by its necrosing effect upon the tissues." Notwithstanding the most pronounced expressions of those best qualified to determine upon the injurious effects of irrigation with mercuric solutions, it is found that some members of the medical profession continue to use them from force of habit, and they must learn by bitter experience to abandon the error of their ways.

The elementary and fundamental question in regard to the animal or vegetable nature of the germs of disease, occupied bacteriologists for many long years; and though it has been finally accepted, as the established doctrine, in favor of germs of vegetable origin, we cannot rely implicitly upon the results of this investigation. The division of micro-organisms into the coccus, bacillus and spirillum, gives the starting point for a multitude of subdivisions; and the vocabulary is constantly increasing as new workers come into the field. A recent writer thinks it may eventually become possible to neutralize the effect of the most deadly microbe by generating a variety inimical or destructive to it. By this achievement, bacteriology is claimed to have taken a new departure, of which it is impossible to forecast the consequences in the future.

It is evident that many elaborate disquisitions on this subject are not based upon a personal knowledge of the facts, and the authors run into inconsistencies in explaining the outgrowth of their theories. It may turn out that my hearers will place the present writer in the same category before reaching the end of this paper.

The relations of bacteria to diseased processes in the organism depend to a very large extent upon the predisposition of the structures to take on disease, and upon the development of leucocytes which arrest the morbid element. It is held generally by bacteriologists that the predominance of phagocytes in the tissues, which may be involved, prevents the hurtful influence of bacteria and that the phagocytal process serves as a prophylactic against structu ral disorders.

Indeed it is questionable whether active living micro-organisms cause disease, and it may be fairly inferred that the loss of vitality in the micrococci is the source of trouble in most cases of morbid developments.

This lends force to the view that ptomaines have an important role in the disorders of the physical organization, and the impression has been growing that this is the septic element in the suppurative and destructive results of any contamination from without. It is evident that ptomaines contribute chiefly to the development of septicæmia in most cases.

A ptomaine is defined by Foster to be "an alkaloid formed as the result of putrefaction or abnormal fermentative changes taking place in an organ after death or as a consequence of morbid action during life. Ptomaines are probably in all cases due to the decomposition of putrid or other organic compounds by bacterial action. Some ptomaines are poisonous, others are physiologically inert."

According to the same authority, Encyclopaedic Medical Dictionary, septicæmia is a constitutional, generally acute disease, popularly called blood-poisoning; due to the absorption of various putrid substances into the blood, which are supposed to act as ferments and so to change it that it cannot fulfill its physiological functions. The lymphatic infection is a form of septicæmia in which the infecting material has entered the circulation by way of the lymphatics; it is rapid and severe in its course and characterized by effusions in the several cavities. Peritoneal septicæmia is a form that may occur and prove rapidly fatal in consequence of a wound of the peritoneum with or without peritonitis. Puerperal septicæmia is due to septic infection in the puerperal state. Venous septicæmia is that in which infection proceeds from a putrid thrombus; as, in uterine phlebitis.

The most serious form of septicæmia which is encountered in surgery is that in which the toxic influences extend to the nerve centers with but little local manifestations in the tissues involved, excepting the loss of vitality. This overpowering impression prevents the ordinary inflammatory developments in the adjacent structures and instead of finding hyperæmia of the area involved, a necrosed condition is observed, which is speedily followed by general collapse with subnormal temperature. This insidious progress of the toxæmia may escape attention at the outset and only become notable by the gravity of the symptoms indicating an early fatal termination.

It may be inferred in these cases that such a transformation is produced by the development of ptomaines in the part affected, that mortification ensues; and although the patient does not suffer pain, this lethal impression is propagated through the nerve centers to the vital organism.

While stimulants of the most energetic character are indicated under such circumstances, they are found insufficient to avert the death of the patient.

In that class of septic manifestations, known as dissection wounds, the absorption of the decomposed elements from a dead body presents in a typical form that quality attributed to ptomaines. The word implies a concentration of malignant death-dealing energy; and its destructive force is exhibited by the smallest appreciable particle of the ptomaines which may enter into the organism. It possesses a most extraordinary property of a toxic nature, which diffuses itself through the lymphatics, and results in the disorganization of remote as well as neighboring structures. The most serious consequences ensue from the slightest abrasion of the cuticle, which admits of absorption of the toxic element, and it is rare that its progressive work of destruction can be arrested without disintegration of the tissues involved. Those who witnessed the influence of dissection wounds before the introduction of antiseptic injection of dead bodies, need not to be reminded of the grave results, and those who incidentally have met with instances in latter days, must be profoundly impressed with its terrible agency for disorganization of vital structures.

We are informed that "pyæmia usually follows wounds, suppurative inflammation of bone, on the puerperal state; and results in the formation of secondary abscesses in the viscera joints and connective tissue; and that it is sometimes associated with phlebitis or embolism." But this term is only applicable to those cases in which pus enters into the calculation. The morbid effects from other sources may be designated as toxæmia, being the result of some toxic agent of a different kind, but should not be included in the category of pyæmia. Thus it turns out that the cases of blood-poisoning may be numerous in which the element of purulent infection does not exist. It is true that cases of pyæmia without any discoverable lesion have been reported, and it is hence inferred that the condition may occur as an idiopathic disorder. But in this as in tetanus, the weight of evidence is in favor of a local origin, and it is in many instances difficult to detect the focus of suppuration. The development of pyæmia cannot take place without the previous formation of pus, which becomes absorbed by the lymphatics.

The studies of Virchow concerning thrombosis and embolism, showed that the changes in the veins which had been regarded as due to phlebitis were caused by the coagulation of the blood, and by subsequent degenerative changes in the thrombi thus formed; that the infarction and abscesses seen in the veins were due to emboli which had become detached from the softened thrombi, that as the white blood globules and pus globules were identical in appearance they could not be distinguished, and that it was improbable that pus globules found their way into the blood. On the other hand Sedillot, Weber, Billroth and others have held that laudable fresh pus was capable of being absorbed and of producing a febrile movement (International Encyclopaedia of Surgery). In support of the view that the poison might be one of the chemical products of putrefaction Panem's investigation led to the conclusion "that there is in putrifying fluids a specific chemical substance, soluble in water. This substance, if introduced into the blood, produces the peculiar symptoms which belong to what is usually called putrid or septic infection. This possesses such properties after being freed from aicroscopic organisms."

It is no longer held that pus enters bodily into the circulation except in the event of ulceration of the venous walls, but the contact of pus with the lymphatics, leads by a pathological process to septic absorption and hence to general contamination of the system. The experiments which have been resorted to for recognizing a distinction between the white corpuscles of the blood and pus corpuscles have not afforded definite results, nor has the hypothesis of a third blood corpuscle availed anything towards a solution of the problem connected with purulent infections.

The fact of a toxic influence being conveyed by pus through the lymphatics to the vascular system is recognized generally by pathologists, and while the link which connects cause and effect has not been clearly established, the clinical relation is sufficiently definite for all practical purposes. The collection of pus in the internal organs when confined by a pyogenic membrane may develop hectic fever, which disappears after the evacuation of the abscess, but the removal of a purulent focus of absorption does not always arrest pyæmia.

That pyæmia may occur independent of any ptomaine element is a fair inference from the history of cases in which purulent absorption seems to account satisfactorily for the train of disorders which are observed. In the cases of multiple abscess which are developed from a purulent focus, there is no reason to doubt that pus has entered the lymphatic system, and, being arrested at certain points, leads to local collections of a purulent character. These abscesses conform in most respects to the characteristics of what is known as cold abscess, partaking of the nature of chronic abscesses; and are rarely accompanied with the acute inflammatory manifestations of phlegmonous abscesses.

When a purulent focus leads to the absorption of pus there must be a deficient formation of that protecting sac known as the pyogenic membrane, and, consequently, the purulent collection escapes into the collective tissue, and is taken up by the lymphatics and conveyed to the blood vessels or the capillaries. It then becomes absorbed and, in some way [not very well defined, develops pyæmia, commonly understood as blood-poisoning.

There may exist a local contamination from contact with septic matter, which spends its influence upon the part and forms a circumscribed abscess, without manifesting constitutional effects. Again there may occur absorption of pus through a slight prick or abrasion of the cuticle, attended with but little local irritation, which shall lead to general disturbances of a grave character. The development of lymphangitis is marked by redness and tenderness along the tract of the lymphatics and with inflammation of the lymphatic glands. This is accompanied with decided febrile reaction and with prostration of the vital forces, terminating either by resolution of the inflammatory process or by suppuration at different points from arrest of the lymph in the swollen ganglions, It sometimes appears to lessen and mitigate the constitutional disturbance when these local collections of pus are discharged by free incision and drainage. But on other occasions no relief is afforded by their evacuation, and other points become the seat of suppuration with an aggravation of the symptoms of purulent absorption and infiltration.

These collections of pus are not confined in a distinct and circumscribed area by a pyogenic membrane, but permeate the cellular tissue and extend along the sheaths of the tendons, thus assuming the character of a diffused abscess. The toxæmia may be limited to one side of the body entirely or may extend throughout the absorbents on both sides and thus become general, involving the whole system. The exhaustion of the vital forces at this stage is usually accompanied with great emaciation, and the nutritive process becomes so much impaired that nourishment avails but little. In a paper submitted for consideration of the American Surgical Association in 1893, the full report of a most protracted case of pyæmia was given in detail. This patient presented all the various features of the different stages of this affection and I would refer those who are interested in the history of this disorder to the transactions of that year for information in regard to the progressive destructive work of this disease to its fatal termination after the lapse of nearly five months, attended with extreme emaciation.

As an offset to this fatal result, I may advert to two fortunate cases of great gravity in members of the medical profession—one being in the person of an interne in the Providence Infirmary, at Atlanta; and the other being that of a prominent practitioner, of Anniston, Ala.

The former occurred from slight injuries to the hand in making a *post-mortem* examination, under my observation, and came under my care, beginning with points of local irritation, extending to the entire arm, and becoming, in a few days, a constitutional disorder. It ran through a most violent course, attended with suppuration at various points in the hand and arm, and leaving the subject extremely prostrated and emaciated, with a long, protracted convalescence. There was finally remaining only the stiffening of two fingers of the affected hand.

The latter case, above mentioned, was seen in consultation with colleagues, and was in consequence of a scratch by a piece of necrosed bone, in performing a surgical operation. Incisions had already been made to relieve tension at several points about the hand and wrist, and the patient was kept under the influence of morphine. It may be briefly stated that with the treatment, which will be outlined, he eventually recovered entirely.

The indications at the outset, when there is local irritation at the point of injury, is to incise the affected part, and cauterize with carbolic acid, applying to the adjacent area an ointment containing equal parts of the following:

℞. Ung. Hydrarg.
Ung. Iod. Comp.
Ung. Camph.
Ung. Bellad.

A poultice of flaxseed meal may cover the inflamed structure, and be renewed frequently, to keep up heat and moisture.

If there should follow much swelling and great tension, incisions should be made freely to relieve the constriction, and give an outlet to the sero-sanguinolent accumulations. This course is more especially indicated when the hand is involved; and when the inflammation extends up the arm, invading the axillary glands, the ointment and poultice should be carried up to the shoulder, so as to cover the entire arm.

If the case progresses favorably, there may be no accumulation of pus above the elbow, but if abscesses form, they should be promptly evacuated, and washed out with antiseptic solutions.

It may be laid down as a general rule for our guidance, that any original or secondary purulent focus should be freed from septic matter, in whatever form it may exist. If not accessible by curetting or swabbing out, then we may avail ourselves of syringing with those antiseptic solutions which are least likely to do harm by absorption, such as peroxide of hydrogen, permanganate of potash, and Lugol's compound solution of iodine. In the event that blood clot has remained in the womb after labor or abortion, and is undergoing decomposition, there is, in my observation, a decided preference for a strong solution chloride of sodium, as it conduces best to dissolving the blood clot. This holds good in the case of sanguineous deposits in the abdominal cavity, in hæmatoma wherever found, and in the accumulation of menstrual fluid, though there may be no coagula to dissolve, and whenever a septic process may result from the collection of blood in the tissues. The solutions of carbolic acid and corrosive sublimate should not be used, as by absorption, they may prove detrimental.

The medicinal treatment must be varied, according to the condition of the general system at the different stages of infection, and at the outset, calomel and Dover's powders in moderate doses, followed by Epsom salts, with senna tea, are usually indicated. After free purgation, it is found that quinine and phenacetine have a most salutary effect, and if there is great suffering with pain and restlessness, morphine and atropia may be employed hypodermically to secure rest.

To modify acute inflammation before the suppurative stage, the combination of fluid extract of jaborandi with salicylate of soda, has been attended with most salutary effects, and should be urged to the extent of tolerance. It is to be expected that free diaphoresis and profuse salivation will eliminate to a large extent the poisonous contamination of the vascular system.

I had occasion to observe some years ago, a most happy and complete transformation in a case of puerperal septicæmia from the subcutaneous injection of one-tenth grain of pilocarpine which produced profuse sweating and relieved the patient of all septic troubles.

After the development of abscesses the chief reliance is upon tonics and depuratives; my experience with the following combination has been quite satisfactory:

℞. Tinc. Cinchonæ Comp. f℥ii.

Tinc. Nucis Vomicæ, fℨi.

Kali Chloratis, ℨi.

Aquae, q. s. f℥vi.—M.

Sig. Tablespoonful every three hours.

When the toxæmia progresses in connection with the purulent discharge it has appeared to be advantageons to introduce into the system gradually an alterative and tonic, such as the following:

℞. Donovan's Solution, fℨiv.

Tinc. Gentian, f℥iiiss.—M.

Sig.: Teaspoonful every four hours.

To arrest disintegration of structures, various preparations of iron, such as the albuminate of iron, iodide of iron and the muriated tincture, with cod liver oil, lager beer, malt, etc., have been used with benefit in the advanced stages. Everything which can aid the recuperative process is indicated.

The proper sphere of real antiseptic surgery comes into play with pyæmia and septicæmia, as these are pre-eminently septic developments which require the use of measures to counteract the contamination of the organism. Not only in the local but in the general manifestations is sepsis to be combated, and hence it becomes requisite to correct the secretions and stay the disease of the organs by internal antiseptic remedies. Some have regarded alcoholic treatment as indicated from the outset of the disorder, and that nourishment of a character to prevent the waste of tissues should be combined with the alcoholic stimulant. The mixture of milk and whiskey or brandy, in form of milk-punch, is found to meet the requirements satisfactorily as a routine diet. But it is desirable to supplement this with beef soup, corn meal gruel, malt and fresh vegetables, such as Irish potatoes, ochra and tomatoes, suited to the state of the digestive organs and to the power of assimilation. It is a matter of pre-eminent importance to sustain the physical organization throughout the septic process.

